# Optimizing a Technology-Based Body and Mind Intervention to Prevent Falls and Reduce Health Disparities in Low-Income Populations: Protocol for a Clustered Randomized Controlled Trial

**DOI:** 10.2196/51899

**Published:** 2023-10-03

**Authors:** Ladda Thiamwong, Rui Xie, Joon-Hyuk Park, Nichole Lighthall, Victoria Loerzel, Jeffrey Stout

**Affiliations:** 1 Nursing Systems Department College of Nursing University of Central Florida Orlando, FL United States

**Keywords:** fall prevention, fear of falling, low income, older adults, exercise, technology

## Abstract

**Background:**

The lack of health care coverage, low education, low motivation, and inconvenience remain barriers to participating in fall prevention programs, especially among low-income older adults. Low-income status also contributes to negative aging self-perceptions and is associated with a high perceived barrier to care. Existing fall prevention intervention technologies do not enable participants and practitioners to interact and collaborate, even with technologies that bring viable strategies to maintain independence, prevent disability, and increase access to quality care. Research is also limited on the use of technology to enhance motivation and help individuals align their perception with physiological fall risk. We developed a novel, 8-week Physio-Feedback Exercise Program (PEER), which includes (1) technology-based physio-feedback using a real-time portable innovative technology—the BTrackS Balance Tracking System, which is reliable and affordable, allows for home testing, and provides feedback and tracks balance progression; (2) cognitive reframing using the fall risk appraisal matrix; and (3) peer-led exercises focusing on balance, strength training, and incorporating exercises into daily activities.

**Objective:**

This study consists of 3 aims. Aim 1 is to examine the effects of the technology-based PEER intervention on fall risk, dynamic balance, and accelerometer-based physical activity (PA). Aim 2 is to examine the effects of the PEER intervention on fall risk appraisal shifting and negative self-perceptions of aging. Aim 3 is to explore participants’ experiences with the PEER intervention and potential barriers to accessing and adopting the technology-based PEER intervention to inform future research.

**Methods:**

This is an intention-to-treat, single-blinded, parallel, 2-arm clustered randomized controlled trial study. We will collect data from 340 low-income older adults at baseline (T1) and measure outcomes after program completion (T2) and follow-up at 3 months (T3) and 6 months (T4). Participants will be enrolled if they meet all the following inclusion criteria: aged ≥60 years, cognitively intact, and able to stand without assistance. Exclusion criteria were as follows: a medical condition precluding exercise or PA, currently receiving treatment from a rehabilitation facility, plan to move within 1 year, hospitalized >3 times in the past 12 months, and does not speak English or Spanish.

**Results:**

As of August 2023, the enrollment of participants is ongoing.

**Conclusions:**

This study addresses the public health problem by optimizing a customized, technology-driven approach that can operate in low-resource environments with unlimited users to prevent falls and reduce health disparities in low-income older adults. The PEER is a novel intervention that combines concepts of physio-feedback, cognitive reframing, and peer-led exercise by motivating a shift in self-estimation of fall risk to align with physiological fall risk to improve balance, PA, and negative aging self-perception.

**Trial Registration:**

ClinicalTrials.gov NCT05778604; https://www.clinicaltrials.gov/ct2/show/study/NCT05778604

**International Registered Report Identifier (IRRID):**

DERR1-10.2196/51899

## Introduction

### Background

In 2020, there were 37.2 million people living in poverty in the United States, approximately 3.3 million more than in 2019 [1]. Every 19 minutes, an American individual dies from a fall, and every 11 seconds, an older individual is treated in an emergency department for a fall [2]. Falls and fear of falling (FOF) are significantly higher in racially diverse low-income older adults than in the general older population [3-5]. There is an association between poverty, fall injury, and mortality, resulting in health disparities [6,7]. Falls cause 95% of hip fractures, 40% of admissions to facility-based care, and 40% of fall victims to lose mobility and independence [8]. In 2018, the direct costs of fatal falls were >US $754 million, and nonfatal falls were US $50 billion, but only US $9 billion was paid by Medicaid [9]. As the number of low-income older adults increases sharply and inequalities continue to grow, this is now a widely recognized problem that requires urgent and significant action [10]. The lack of health care coverage, low education, low motivation, and inconvenience remain barriers to participating in fall prevention programs [11,12]. Low-income status contributes to negative aging self-perceptions [13] and is associated with a high perceived barrier to care [14]. A higher level of concern over falling and FOF were more prevalent in low-income older adults and functional independence in daily activities [12]. Low-income status is independently associated with FOF, which induces activity restriction [15] and functional decline [16]. People who live in low-income communities are less likely to engage in physical activity (PA) [17-19]. The lack of PA is related to falls and poor quality of life [20,21].

Addressing maladaptive fall risk appraisal (FRA) can be challenging owing to self-report bias and cognitive deficit. We developed an *FRA matrix*, a graphical grid categorizing perception (levels of FOF) and body function (level of balance) into four groups: (1) rational FRA (low FOF and normal balance), (2) incongruent FRA (low FOF despite poor balance), (3) irrational FRA (high FOF despite normal balance), and (4) congruent FRA (high FOF and poor balance) [22,23]. On the basis of our previous works [24-26] and other studies [27], approximately 64% to 79% of older adults had maladaptive FRA. Older adults in maladaptive FRA groups were 2 to 3 times more likely to fall than those in adaptive FRA [24,25]. Those with irrational FRA serve as a barrier, creating a high FOF that inhibits low-income older adults from staying physically active. High FOF restricts mobility and daily activities and increases fall risk [28], with chronic FOF predicting an increased risk of functional decline [16]. Individuals with FOF overestimated their gait performance and showed deficits in motor imagery of gait [29]. They may not exhibit increased FOF and subsequently fall if they accurately appraise their physical abilities [27]. Evidence, including our data, indicates that an intervention program must help older adults reframe FRA and maintain PA [27,30]. We found that the FRA matrix is associated with moderate to vigorous PA (MVPA), Centers for Disease Control and Prevention (CDC) fall risk score, self-reported strength and flexibility, difficulty walking several city blocks, and left-hand average handgrip strength [31].

Older adults with FOF overestimated their gait performance and showed deficits in motor imagery of gait [29]. Older adults may exhibit less FOF and subsequently fall if they accurately appraise their physical abilities [27]. In addition, maladaptive FRA can result in activity withdrawal, decline in physical and cognitive abilities, and increased social isolation [23]. Therefore, adaptive FRA is a key component of activity participation and maintenance of self-reliance [32]. Several types of interventions focus on reducing FOF [33-41]; however, some degree of FOF can increase conscious awareness and reduce fall risk by encouraging individuals to avoid exposure to unnecessary risks [42]. Low-income older adults with better-perceived physical health may experience less FOF [43].

In addition, existing fall prevention intervention technologies do not enable participants and practitioners to interact and collaborate [44] even with technologies that bring viable strategies to maintain independence, prevent disability, and increase access to quality care. Acceptability, privacy issues, technology costs, implementation costs, and barriers have rarely been addressed [44]. Research is also limited on the use of technology to enhance motivation and help individuals align their perception with physiological fall risk [45]. Offering additional advantages, technology-based exercise interventions have good adherence and may provide a sustainable method of promoting PA and preventing falls [45,46].

We developed a novel, 8-week Physio-Feedback Exercise Program (PEER) [32], which includes (1) technology-based physio-feedback using a real-time portable innovative technology—the BTrackS Balance Tracking System (BBS), which is reliable [47,48] and affordable, allows for home testing, provides feedback, and tracks balance progression; (2) cognitive reframing [49] using the FRA matrix; and (3) peer-led exercises [50] focusing on balance, strength training, and incorporating exercises into daily activities. Our pilot study supports the feasibility and acceptability of using BBS technology in low-income communities to screen individuals with maladaptive FRA. It also supports using the results to tailor interventions to improve PA and reduce falls [31,51,52]. The findings highlight reducing sedentary time to help shift from irrational to rational groups and increasing MVPA time to help shift from congruent and irrational to rational FRA groups. We found a significant difference in sedentary time between the rational and irrational groups (*P*=.04). This indicates that if a person exhibits high FOF despite their normal balance (irrational), they tend to be more sedentary compared with people with low FOF and normal balance (rational). Importantly, we showed that the FRA matrix and 4-group quadrant design (irrational, incongruent, congruent, and rational) allows stratified analysis, which provides a more efficient overall fall risk assessment, which has rarely been reported in the literature.

### Aims and Hypotheses

Aim 1 is to examine the effects of the technology-based PEER intervention on fall risk, dynamic balance, and accelerometer-based PA.

The hypotheses are as follows:

- *H 1.1:* The PEER group will reduce the fall risk compared with the control group.
- *H 1.2:* The PEER group will improve the dynamic balance compared with the control group.
- *H 1.3:* The PEER group will improve accelerometer-based PA compared with the control group.

Aim 2 is to examine the effects of the PEER intervention on FRA shifting and negative self-perceptions of aging.

The hypotheses are as follows:

- *H 2.1:* The PEER group will have a more adaptive shifting compared with the control group.
- *H 2.2:* The PEER group will have a more positive shift in their perceptions of aging compared with the control group.

Aim 3 is to explore participants’ experiences with the PEER intervention and potential barriers to access and adoption of the technology-based PEER intervention to inform future research. A purposive sample of 30 participants from the PEER group will participate in semistructured one-on-one interviews to explore their perceptions of barriers to access and adoption of technology and intervention.

## Methods

### Study Design

This is an intention-to-treat, single-blind, parallel, 2-arm clustered randomized controlled trial study. We will collect data at baseline (T1) and measure outcomes after program completion (T2) and follow-up at 3 months (T3) and 6 months (T4) to test our hypotheses. We include a retention period of 6 months to determine whether fall risk, dynamic balance, PA, altered maladaptive FRA (FRA shifting), and negative self-perceptions of aging are maintained without ongoing intervention. This study is registered on ClinicalTrials.gov (NCT05778604).

### Settings

The intervention will be offered in low-income, independent living communities, units, and apartments in Orlando, Florida. Florida has a large aging population with drastically different income levels and degrees of socioeconomic status [53]. The diversity of potential participants is also high, as Florida’s population is approximately 17% African American, 3% Asian, 26% Hispanic or Latinx, and 54% non-Hispanic White [54].

### Study Participants

A sample of 340 participants will be enrolled if they meet all the following inclusion criteria:(1) aged ≥60 years, (2) cognitively intact based on Mini-Mental State Examination score ≥24 [55], and (3) able to stand without assistance. Exclusion criteria were as follows: (1) a medical condition precluding exercise and PA, such as feeling pressure when performing PA; (2) currently receiving treatment from a rehabilitation facility; (3) plan to move within 1 year; (4) hospitalized >3 times in the past 12 months; and (5) does not speak English or Spanish.

### Power Analysis and Sample Size

The sample size estimate was based on the primary outcomes of fall risk (CDC fall risk score) and PA (normalized MVPA) for aim 1 and the secondary outcome of FRA shifting (BBS score) for aim 2. The difference in the normalized mean MVPA between the PEER group (mean 3.67, SD 2.54) and the control group (mean 5.38, SD 2.68) and the difference in the fall risk score between the PEER group (mean 2.72, SD=3.23) and the control group (mean 2.21, SD=2.06) are considered markers of PA and fall risk, respectively. Considering a clinically relevant normalized difference in MVPA of 1.7 (SD 2.60) and a fall risk score difference of 0.50 (SD 2.56), the medium effect size Cohen *d*=0.25, intraclass correlation (ICC) of 0.8, and a significance level of are used for the calculation of the sample size. A sample size of 120 participants per arm is needed to reach 80% statistical power to detect the difference between the PEER and control groups on repeated measures of normalized MVPA. A sample size of 120 participants per arm is needed to reach 80% statistical power to detect the difference between the PEER and control groups on repeated measures of normalized MVPA.

In addition, the sample size was also based on the following: (1) at least 3 sites per arm; (2) an initial hypothesis that at least 10% (34/340) of the irrational, incongruent, and congruent FRA would shift to rational FRA after PEER completion; (3) an approximate study adherence rate of 80% [56], and (4) an estimated study dropout rate of approximately 40% (136/340) over time. Therefore, the sample size was increased from 120 to 170 per arm, resulting in a total of 340 participants. On the basis of our pilot study [26], we estimate that approximately 60% (204/340) of the low-income older adults will meet all inclusion and exclusion criteria and will be interested in participating in this study. We estimate that a minimum of 567 low-income older adults will be screened to identify 340 low-income older adults for enrollment, and approximately 240 low-income older adults (120 per arm) are needed to complete the study. Given the sample size of 120 per arm, a significance level of *P*=.05, and 80% power, considering a clinically relevant BBS mean score difference of 8 (SD 12), the effect size Cohen *d*=0.36 can be achieved to compare the intervention effect based on secondary outcomes.

### Recruitment, Randomization, and Procedures

Research sites will be recruited through a partnership with local communities, personal contacts, referrals, and phone calls. The unit of randomization is an independent living senior community, which is defined as a company-owned facility with a design for low-income independent adults aged ≥55 years and without health care services provided on campus. Research sites will be selected based on the prespecified criteria, including the number of residents, geographic proximity (urban vs suburban), and ability to implement the intervention. We plan to recruit 6 sites (3 intervention sites and 3 control sites). Collectively, approximately 10 sites and 800 low-income individuals are available for screening, and we can add sites, as they are abundant in the area. The participating sites will be randomized to either the intervention or control group in a 1:1 ratio. RX (coinvestigator and statistician) will use a computerized pseudorandom number generator to determine the randomization order in advance. The project director (PD) will reveal the randomization of the sites before the study [57]. Following randomization, we will recruit participants using standard strategies, including placing flyers on information boards and participating in open-house sessions or health fairs for face-to-face recruitment. Participants will also be recruited from their units by email. We will screen participants by a phone call to determine their initial eligibility, and those eligible will be invited for the baseline assessment. The research staff will explain the study and complete the consent process. The PEER intervention sites will be required to select 6 to 7 volunteers called *peer coaches* (PCs) to lead the exercise group, and the PCs will be eligible if they report having a regular PA routine or having an educational background in health care; are aged >55 years; can read, write, and understand English and Spanish; and are committed to delivering the protocol for 8 weeks. JS (coinvestigator) and the principal investigator (PI) will provide two, 3-hour sessions to train the PCs in balance and strength training, safety in the exercise, and participant motivation in collaboration with a successful PC from our pilot study. Each PC will be matched to 8 to 10 low-income older adults in the PEER group according to the place of residence and will supervise low-income older adults in groups while they are engaged in group exercises. This procedure was successful in our pilot study.

### Intervention

The PEER consists of 3 components with 3 steps.

#### Step 1: Technology-Based Physio-Feedback

**Figure 1:**
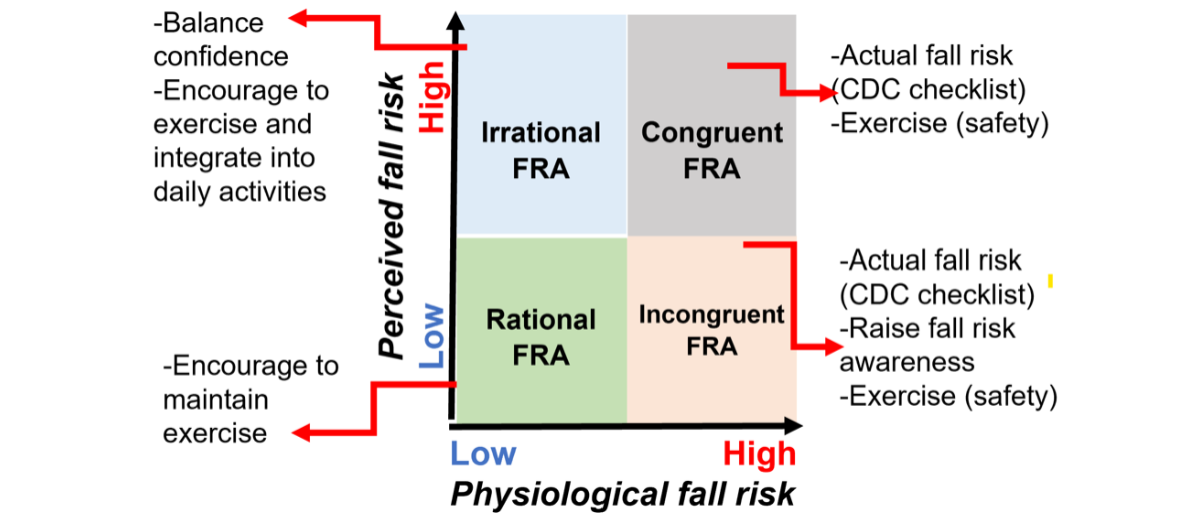
Technology-based physio-feedback. CDC: Centers for Disease Control and Prevention; FRA: fall risk appraisal.

The research assistant (RA) presents the BTrackS software (Balance Tracking Systems, Inc) to display the participant’s BBS scores with interpretations and then categorizes the participants into 4 groups (irrational, incongruent, congruent, and rational) according to the FRA matrix and plots the position in 1 of the 4 quadrants on the FRA matrix chart (Figure 1). The RA will provide physio-feedback to the participants at baseline (T1) and after program completion (T2).

#### Step 2: Cognitive Reframing

*Cognitive reframing* will be based on the FRA matrix. We will present the FRA matrix, and activities will be tailored based on the quadrant that the participant fits (Figure 1):

- *Quadrant 1* (irrational): focus on increasing balance confidence and maintaining exercise.
- *Quadrant 2* (incongruent): focus on individual fall risk factors from the CDC fall risk checklist, enhance fall risk awareness, and participate in the exercise.
- *Quadrant 3* (congruent): focus on individual fall risk factors from the CDC fall checklist and participate in the exercise.
- *Quadrant 4* (rational): encourage to maintain exercise.

#### Step 3: Peer-Led Exercises

*Peer-led exercises* focus on balance, strength training, and incorporating exercises into daily activities. A trained PC will lead the group-based exercises for 60 minutes (8-10 per group). Exercise training includes 4 sets of warm-ups, strength for the upper and lower body, balance (standing and moving), and stretching of the upper and lower body. Our training sessions follow the guidelines established by the American College of Sports Medicine, which take into account the frequency, intensity, time, and type of exercise recommended for each individual level of progression. These levels are classified as beginner, intermediate, and advanced [58,59]. On the basis of these guidelines, a duration of 91 to 120 minutes of exercise per week is the most effective in improving overall balance performance [60]. Therefore, the dose of exercise that will be tested in the PEER group is 120 minutes for 8 weeks (60 min/wk for group exercise led by the peer and at least 30 min twice a wk for individual exercise). We will offer a booklet with exercise instructions in both English and Spanish, featuring illustrations to guide users through each exercise set. The booklet contains a diverse range of exercises that can be easily incorporated into daily routines, such as cooking. The participants will be instructed to keep a weekly log of their exercise activities, noting the type and duration of each activity they performed at home.

### Treatment Fidelity and Monitoring

The PI and PD will oversee the treatment fidelity and monitoring plan. We will use a modified Treatment Fidelity Assessment Tool based on National Institutes of Health’s Behavioral Change Consortium treatment fidelity research [61]. In total, 5% (17/340) of the intervention sessions will be randomly observed by the PI using the modified Treatment Fidelity Assessment Tool, including treatment design, training, delivery of treatment, receipt of treatment, and enactment of treatment skills [61,62]. It is evaluated on a 3-point scale (1=present, 2=absent but should be present, and 3=not applicable). Problems identified, including deviation from the protocol, will be discussed with the research team, assessors, and interventionists biweekly. Retraining will be provided if adherence is <90%. A continual process evaluation plan will be used in this study. The plan is based on the guidelines of the Medical Research Council framework [63], which includes three components: (1) implementation, (2) mechanisms of impact, and (3) contextual factors. The implementation of the PEER intervention will be evaluated based on fidelity (whether the PEER was delivered as intended), completeness (quantity of the PEER implemented), adaptation (alterations made), and reach (characteristics of the population reached). The mechanisms of impact (how the delivered PEER produced change) and contextual factors (how context affects implementation and outcomes) will be assessed in this study. Adherence to the PEER intervention will be monitored by phone calls by a PC. Adverse events will be monitored throughout the study period. Low-income older adults will be instructed to contact RAs, the PI, or the PD if they have falls, near falls, or a change in status that led to medical attention. Contact will be recorded by the RAs. The low-income older adults will be asked to identify reasons for adherence as well as nonadherence. A project logbook, registration checklists, participant records, and self-reports will be used to collect these data.

### End Points

The intervention will be delivered per protocol until 1 of the following end points is met (whichever occurs first): (1) unable to participate in PEER activities (eg, exercise), (2) past 8 weeks of enrollment, and (3) other exclusion criteria are met (eg, severe cognitive problems).

### Control Group

The control group or enhanced usual care will receive an information pamphlet about falls (English or Spanish version) developed by the CDC Stopping Elderly Accidents, Deaths, and Injuries–Older Adult Fall Prevention. These pamphlets provide details on fall risk, ways to prevent falls, how to check for safety, postural hypotension, chair rise exercise, what they can do to avoid falls, ensure safety, and manage postural hypotension [2]. The control group will be encouraged to discuss fall prevention with their primary care provider and continue their regular activities for 6 months. They will be offered the PEER intervention when the study concludes. They will receive monthly phone calls from the RA to record a monthly fall incidence log.

### Outcome Measures and Data Collection

#### Primary Outcome Measures

##### Fall Risk

*Fall risk* will be assessed using the CDC’s Stopping Elderly Accidents, Deaths, and Injuries fall risk checklist. It consists of 12 statements related to physical and psychological fall risk factors, with responses of either yes or no. If a person scores ≥4 points, it suggests that there is a risk of falling [2]. The sensitivity of this checklist for discriminating fallers and predicting future fallers among community-dwelling older adults is 73% to 80% [64].

##### Dynamic Balance

*Dynamic balance* will be assessed using the “Timed Up and Go” (TUG) and “Sit to Stand” (STS) tests suggested by the CDC [2] and the American Geriatric Society [65]. It is important to conduct >1 balance test, as some participants may be identified as at risk on one and not another [64]. The TUG test is a commonly used method for evaluating functional mobility and predicting the risk of falling [66,67]. The TUG test has been validated among older adults in the community [68]. The TUG test has been proven to be effective in assessing older adults at risk of falling, with a sensitivity and specificity of 87% [69]. The STS is reliable for various populations [70,71]. For the TUG test, participants will stand up from a standard armchair, walk at a normal pace for 3 m, return, and sit down again [72]. Participants who complete the TUG test in <12 seconds will be classified as having a low fall risk [73]. Then they will complete the STS. To begin, the participant should sit in the center of the chair, crossing their hands at the wrists and placing them on the opposite shoulder. They should keep their feet flat on the floor, their back straight, and their arms against their chest. Then, they should repeat the motion of standing up and sitting back down for 30 seconds. The results will be scored based on their age and sex as suggested by the CDC [2].

##### PA Measures

*PA* participants will have their activity levels monitored using ActiGraph GT9X Link wireless activity monitors for 7 consecutive days. These devices, which are triaxial accelerometers, will be worn on the nondominant wrist by all participants. The GT9X Link has a sample rate of 30-100 Hz and a dynamic range of ±8G. It also has a rechargeable battery that lasts up to 14 days and can store up to 4 GB of data for 180 days. Information is collected every minute. A sensor checks if the device is being worn or not. The GT9X Link gives accurate 24-hour measurements of PA, such as steps taken, energy expended, intensity, and the user’s position. Measuring PA through accelerometry is a reliable method (ICC=0.98), especially when assessing free-living individuals [74]. It has been validated through comparison with measurements of direct observation, energy expenditure, and sedentary behavior [75,76].

#### Secondary Outcome Measures

##### FRA Shifting

*FRA shifting* consists of (1) maladaptive shifting, which is moving from the rational quadrant into any other quadrant, and (2) adaptive shifting, which is moving from irrational, incongruent, or congruent into the rational quadrant [77]. We use the FRA matrix, a graphical grid categorizing levels of FOF (mind) and levels of balance (body) into four quadrants [22,23]: (1) *rational:* low FOF (short Falls Efficacy Scale-International [FES-I] ≤10) and normal balance (BBS ≤30), (2) *incongruent:* low FOF (short FES-I≤10) despite poor balance (BBS>30), (3) *irrational:* high FOF (short FES-I>10) despite normal balance (BBS≤30), and (4) *congruent:* high FOF (short FES-I>10) and poor balance (BBS>30).

##### Levels of FOF

*Levels of FOF* will be assessed using the short FES-I. It is a 7-item self-report questionnaire (English and Spanish version) that provides information on the level of concern about falls for a range of activities of daily living [78]. On a 4-point Likert scale, low-income older adults will be asked to rate their concerns about the possibility of falling when performing 7 activities such as dressing. The scores range from 7 to 28 [79]. Higher total FES-I scores indicate higher FOF [78]. Scores of 7 to 10 indicate low concern about falling, whereas scores of 11 to 28 indicate high concern about falling [80,81]. The short FES-I has been validated in community-dwelling older adults [80]. Cronbach α=.97 and ICC=0.979 among older adults [82].

##### Levels of Balance

Levels of balance will be assessed using the BBS. During the BBS, a piece of sturdy furniture or a standard walker will be placed within the participant’s reach to reduce the risk that FOF will contaminate performance and enable even frail people to participate. Individuals will stand as still as possible on the BTrackS Balance force plate with their hands on the hips and eyes closed [83]. After the test, there are three results: (1) baseline, postbaseline, and percentage of changes; (2) comparison with sex and age group; and (3) levels of fall risk. In comparison with the age group, the software uses the BBS Normative Database to compare the individual with others in their age group. The BBS score is dependent on age and sex but not body size so that the percentile rankings can be determined across various age groups and for men and women separately [84]. A scale from 0 to 100 represents the percentile ranking of the BBS. A score of 0 to 30 indicates a low fall risk (normal balance) [84].

##### Negative Self-Perceptions

*Negative self-perceptions* will be measured using the Brief Aging Perceptions Questionnaire, which consists of 17 items [84]. In the proposed study, we will calculate an overall negative perception score (range 17-85, as in the study by Fawsitt et al [85] and Freeman et al [86]) by summing all negative scales after reverse scoring positive subscales. In our pilot study (N=48), this measure’s Cronbach α was .64.

### Additional Measures of Interest

#### Social Determinants of Health

*Social determinants of health* from the PhenX tool kit include protocols related to demographics or participants’ characteristics including age, sex, education, history of falls, the number of comorbidities, access to health services, health literacy, and access to health technology [87].

#### Depression

*Depression* will be measured using the Patient Health Questionnaire-9. The Patient Health Questionnaire-9 (English and Spanish) is a valid and reliable tool for screening depression in older adults [88,89]. It has high internal consistency (Cronbach α=.89) [88,89]. Participants will score how often each symptom (eg, feeling tired) was present within the last 2 weeks. The total scores range from 0 to 27, with scores ≥10 indicating moderate depression [88].

#### Anxiety

*Anxiety* will be measured using the Geriatric Anxiety Inventory-Short form, which consists of 5 items, investigates 3 dimensions of anxiety (somatic, cognitive, and affective), and is rated on a 4-point Likert scale, ranging from 0 (not at all) to 3 (all the time) [90-92]. It has adequate internal consistency and validity for screening anxiety in older adults [93].

#### Incidence of Falls

We will measure the number of falls and near falls. A *fall* is defined as an unexpected event in which an individual comes to rest on the ground, floor, or a lower level. An *injurious fall* is defined as hospitalization for or receipt of outpatient care because of a fall [94,95]. A *near fall* is a stumble event or loss of balance that would result in a fall if sufficient recovery mechanisms were not activated. At least 2 compensatory mechanisms (eg, unplanned movement of the arms, legs, and trunk tilt) should be activated [96]. The number of falls and near falls will be assessed using a monthly fall log by low-income older adults and follow-up phone calls by RAs.

#### Exercise Adherence

A weekly exercise log is designed from our pilot work to record activity including the types and duration of exercise that low-income older adults performs at home. A weekly exercise log will be handed to the PC at the group exercise in the week after.

### Data Collection

Demographic data (eg, age, sex, living status, and education level), perceived general health, self-report of vision, medication use, urinary incontinence, and the number of falls in the past 6 months will be assessed at baseline to provide context about low-income older adults. A trained RA will perform all baseline and follow-up assessments and will be blinded to the group assignment. Low-income older adults will be instructed not to inform assessors of their group status. All questionnaires and tests (except the PA test) take approximately 60 to 90 minutes to complete, and no risks or discomfort were associated with the balance and PA tests in our pilot studies.

### Data Management and Integrity

Data files will be built on a password-protected computer using REDCap (Research Electronic Data Capture; Vanderbilt University), which will be housed on a secure university server [97,98]. RX (coinvestigator) and RAs will be responsible for data cleaning and will teach the data entry system to the study team members who will be recording data in REDCap. Following an initial audit, the data will be double entered, and files will be matched to verify the accuracy of the data for the first 5 participants, after which a random 5% (17/340) of the total sample will be double checked periodically throughout the study. The data will be systematically examined for out-of-range values and inconsistencies. We anticipate no more than 5% missing values on any 1 item, as data will be collected in person on site and via phone calls. Missing data will be identified and obtained during follow-up interviews or with follow-up contact with participants. We will create an audit trail to identify and correct issues, protect participants’ privacy, ensure confidentiality, and maintain data integrity [99]. If causes of error other than random variation are identified, we will modify or adjust our procedures and train team members as needed.

### Data Analysis

We will collect demographic information from the participants for the study. We will compare baseline variables, such as essential participant characteristics, primary, and secondary end points, to summarize the differences between the 2 groups at the start of the study (T1). For the analysis of categorical data and continuous data, the chi-square test and 2-tailed *t* test will be used. We use R (version 4.1.2; R Foundation) for data analyses, with a significance level of *P*=.05.

Aim 1 is to examine the effects of the technology-based PEER intervention on fall risk, dynamic balance, and accelerometer-based PA.

The hypotheses are as follows:

- *H 1.1*: The PEER group will reduce the fall risk compared with the control group.
- *H 1.2*: The PEER group will improve the dynamic balance compared with the control group.
- *H 1.3*: The PEER group will improve accelerometer-based PA compared with the control group.

Data will be analyzed according to the intention-to-treat principle [100]. We will model the fall risk, dynamic balance, and accelerometer-based PA as longitudinal outcomes consisting of 4 measurements (baseline or T1, after program completion or T2, follow-up at 3 months or T3, and follow-up at 6 months or T4) using the longitudinal linear mixed model (LMM) [101]. The LMM allows all low-income older adults and their available data to be included in the analysis, even for unequal group sample sizes, and the group heterogeneity can be addressed in the variance structure of random effects. It is hypothesized that the PEER group will show improvement in all outcome measures over time. To test hypothesis *1.1*, between-arm differences for fall risk scores, the likelihood ratio test (LRT) [101] associated with LMM will be used from all follow-up assessment visits. Similarly, the LRTs will be used to test hypotheses *1.2* and *1.3* for between-arm differences for dynamic balance and PA, respectively. The CDC-recommended scoring scheme will be adopted to process fall risk, TUG, and STS data [102]. For example, the average score of the number of STS repetitions a person can complete is based on different age and sex groups [102].

Raw acceleration data from ActiGraph will be downloaded and converted to “.csv” files using ActiLife 6 Software (version 6.13.4; ActiGraph LLC). PA data will be processed using the R package *GGIR* (version 2.4-0). *GGIR* R package used to process multiday raw accelerometer data for PA research [103] includes (1) auto-calibration of acceleration signals according to local gravity [104], (2) detection of nonwear time, and (3) calculation of the average magnitude of dynamic acceleration corrected for gravity (ie, Euclidean Norm Minus One) over 5-second epochs and expressed in milli-gravitational units (mg) [105]. Nonwear time and sustained abnormally high accelerations will be imputed using the default settings [105]. The Euclidean Norm Minus One cut-off points will be used to estimate the total time spent in sedentary behavior, light intensity PA, and MVPA in participants.

For missing data treatment, the missing values will be imputed using the method of multiple imputations [106]. The maximum number of missing values within a scale will be based on the guidelines provided by the scale developers or based on a limit of 25% missing values. In the first analysis, all low-income older adults will be included according to their original group assignment [100]. Participants in the PEER group who completed at least 5 of the 8 group-based sessions will be included in the per-protocol analysis. Although every attempt will be made to ensure that low-income older adults complete all assessments, there may be some participant attrition over time. The missing data mechanism is assumed to be missing at random. The dropout patterns or missing data rates for the 2 arms will be examined to assess the differences. When possible, the models should be modified to account for relevant factors such as age, sex, general health perception, and the number of falls in the 6 months leading up to the baseline. To determine the impact of the PEER intervention during follow-up evaluations, the group-by-time interaction term will be incorporated into the model. Effect sizes (Cohen *d*) [107,108] will be calculated based on the estimated means and the pooled SD.

Aim 2 is to examine the effects of the intervention on FRA shifting and negative self-perceptions of aging.

The hypotheses are as follows:

- *H 2.1*: The PEER group will have a more adaptive shifting compared with the control group.
- *H 2.2*: The PEER group will have a more positive shift in their perceptions of aging compared with the control group.

To test hypothesis *2.1* (FRA shifting), the 4 FRA quadrants will be determined by grid coordinates based on 2 continuous level measures (BBS and short FES-I scores). We will classify shifting into 2 types: maladaptive and adaptive shifting. Adaptive shifting is moving from irrational, incongruent, or congruent to rational. The outcome is a percentage of low-income older adults with baseline (T1) in irrational, incongruent, or congruent quadrants who end up in the rational quadrant after program completion (T2). Maladaptive shifting is a movement from rational to any other quadrant, and the percentage of low-income older adults will be calculated. After program completion (T2), the baseline FRA categories (T1) may remain the same or shift to a different FRA category. We will form a 4×4 contingency table for the 4 FRA categories at each follow-up time point (T3 and T4) to summarize the shifts. The standard 4×4 table analysis will be performed to examine the FRA shifting, either using a chi-square test or exact test, depending on the actual counts observed in the 4×4 table. The BBS and short FES-I scores will be collected in 4 planned visit time points (T1, T2, T3, and T3). We will use the Generalized Linear Mixed Model (GLMM) to analyze these 2 end points for assessing the difference between the PEER intervention and control arms on the shift of FRA categories [109]. Our longitudinal data on the PEER effects of different types of shifting on low-income older adults will contain repeated binary measures of the shifting status over time. The binomial GLMM with the logic link will allow us to assess the difference between the 2 arms in FRA shifting, and the associated LRT [101] will be used to test hypothesis *2.1* if a significant adaptive shifting is detected in the PEER group compared with the control group. The GLMM can address the group heterogeneity by specifying the corresponding covariance structure in random effect terms.

To test hypothesis *2.2*, positive shift in their perceptions of aging, the longitudinal mixed model analysis of variance will be used to test the effects of the intervention by comparing the PEER group with the control group from all follow-up assessment visits. Measurements, such as the negative self-perceptions of aging, can be incorporated into the GLMM model to investigate their potential role as mediators of FRA shifting and examine the effects of the intervention.

Aim 3 is to explore participants’ experiences with the intervention and potential barriers to access and adoption of the technology-based PEER intervention to inform future research.

### Rationale

A qualitative approach will allow us to explore the complex, multilevel pathways through how the PEER intervention affect at individual and peer-group levels. We may also identify barriers to successful FRA adaptation and barriers to access and adoption of the technology-based intervention that cannot be identified in quantitative data but will be important to consider in the future. Interviews with participants will provide insights into the variability across settings and implementation factors that will be instructive for program and policy planning. Importantly, interviews will amplify the voices of low-income individuals who traditionally hold less power in program planning [110].

### Design and Sample

We will use in-depth, one-on-one, semistructured interviews with up to 30 participants from the PEER after the completion of the intervention (T2). Semistructured interviews will help ensure that key concepts are addressed while allowing flexibility to adjust and rearrange questions to maximize interview flow and useful data collected. Theoretical sampling will help us gain the perceptions we need in terms of demographic and experiential variation [111,112]. We will sample until saturation of themes and content has occurred. The interviews (30-40 minutes) will be conducted by a trained interviewer either in person or by phone or web conferencing and will be audio recorded. The COREQ (Consolidated Criteria for Reporting Qualitative Research) [113,114] will be used during all phases of the process to optimize quality.

### Interview Guide

The National Institute on Minority Health and Health Disparities research framework [115] will help inform the development of the interview guide and focus on the (1) three domains of influence (biological, behavioral, and sociocultural) and (2) two levels of influence (individual and peer groups) that are most relevant to participant experiences and intervention evaluation. Interview questions will focus on their experience of participating in the PEER intervention, acceptability of PEER, potential barriers to accessing and adopting PEER in the community, and recommendations for future work. Consistent with the standard qualitative methodology, interview guides will be pilot-tested and adjusted based on findings from early interviews [116].

### Analysis

Data will be analyzed based on the analytic process of interpretive description, which is used to develop findings for clinical practice [117,118]. VL (coinvestigator) and the PI will generate initial codes focusing on participants’ experiences with PEER and will meet to establish consensus on the coding and using the NVivo software (version 12; QSR International) to aid in sorting and organizing the data. Consistent with the interpretive description, we will maintain an open stance toward coding to understand participant experiences [118]. Coding will continue until no new information is forthcoming from the data and the categories appear “saturated” [112]. Field notes will be coded to aid data interpretations and provide context, as they will contain interviewers’ impressions and observations during the interviews [111]. We will collate codes into themes, review them for their fit to the data, and categorize and label themes. We will conduct initial analyses on all data as soon as they are collected to allow for theoretical adjustments to the questions and guide sampling strategies for future interviews.

### Domains of Quality

We will enhance quality via the following strategies:

- Maximal variation in sampling by ensuring variability on the factors that likely influence participants’ experience (age and context). We will enhance the credibility of the data and seek out a range of responses to the PEER, both positive and negative.
- Throughout the study, we will document all the methodological decisions and the reasoning behind them to maintain an accurate audit trail and ensure interpretive rigor [111].
- After identifying general themes and subthemes in the qualitative data analysis process, the entire research team will review and discuss a summary through peer debriefing [119].
- We will encourage team members who are not directly involved in the analysis to provide feedback and ask critical questions about the methods, decisions, and interpretation.
- Inference transferability will provide a description of the participants to allow for transferability of findings to similar settings, contexts, and people [120].

### Ethical Considerations

The Institutional Review Board of the University of Central Florida granted ethics approval for this study (protocol STUDY00003206) on October 14, 2022. All participants will receive information before participation.

## Results

As of August 2023, the enrollment of participants is ongoing.

## Discussion

### Principal Findings

Our developed PEER intervention is focused on providing physio-feedback using technology, promoting cognitive reframing, enhancing peer-led exercises, and incorporating exercises into daily activities. We aimed to examine the effectiveness of the technology-based PEER intervention on 3 primary outcomes (fall risk, dynamic balance, and accelerometer-based PA) and 2 secondary outcomes (FRA shifting and negative self-perceptions of aging). We hypothesized that the PEER group will reduce the fall risk, improve the dynamic balance, and improve accelerometer-based PA compared with the control group.

Our pilot study found that the PEER intervention (n=19) had a significant reduction in the CDC fall risk score from preintervention to postintervention (effect size Cohen *d*=0.6; *P*=.02) [32]. We also found that the PEER group had significant improvement in the TUG (*P*=.001; Cohen *d*=1.0) and STS (*P*<.001; Cohen *d*=0.95) tests [32]. In addition, in a pilot study [77], 11% of participants in the PEER group (n=19) had adaptive shifting compared with none in the control group (n=22). Up to 32% of the participants in the control group had maladaptive shifting compared with only 5.3% in the PEER group [77].

Existing fall prevention intervention technologies do not enable participants and practitioners to interact and collaborate [44] even with technologies that bring viable strategies to maintain PA, prevent disability, and increase access to quality care. The physio-feedback using portable technology is a vital component of the PEER intervention for screening fall risk at home or in their communities and initiating a fall risk communication between older adults, their caregivers, and practitioners. The physio-feedback and cognitive reframing strategies improve older adults’ competence by aligning their physiological fall risk with FOF or body and mind and increasing their fall risk awareness [116]. Few studies focused on improving PA in socioeconomically disadvantaged groups [121] despite recommendations for action on the social determinants of health for improving PA. Regular PA improved quality of life and reduced fall risk and mortality [122]; however, a lack of motivation to participate in PA is a crucial barrier. The peer-led exercises with peer coaching strategies may help build a connection and increase the motivation to participate in PA for older adults [50].

### Future Implications

This study addresses the public health problem by optimizing a technology-driven, tailored approach that can operate in low-resource environments with unlimited users to prevent falls and reduce health disparities in low-income older adults. It also supports using the results to tailor interventions to improve PA and reduce falls [31,51,52]. If effective, this intervention can provide an innovative, scalable, and accessible model for fall prevention in diverse and underserved populations. The use of a noninvasive technology that does not require special training and can operate in low-resource environments may scale up the intervention for low-income communities. The BBS can be used for fall risk assessment, tailored interventions, and tracking the changes in balance performance. Technology-based physio-feedback regarding balance has a positive effect on balance confidence and task selection, which could help reframe unrealistic perceptions and lead to healthy behaviors. This study is conducted by an interdisciplinary team and collaborates with community-based and public health organizations, which will ensure the research is relevant, contextually appropriate, and will ultimately be translated into real-world settings.

### Limitations, Potential Challenges, and Alternatives

There are several limitations and potential challenges including (1) recruitment and retention, (2) respondent burden, (3) staffing, and (4) lack of skills to use BBS technology.

#### Recruitment and Retention

Although the recruitment and retention of low-income older adults in randomized controlled trial studies is often challenging, our tailored strategies will address these issues by ensuring that our bilingual RAs are well trained, providing information in English and Spanish versions, and using a straightforward data collection process.

#### Respondent Burden

During the intervention, the PCs and RAs will complete a weekly exercise log and monthly fall or near falls logs. After the 8-week intervention, the only data to be collected monthly from participants will be the fall logs. These were used successfully in our pilot study; we found no falls and 2 near falls in the PEER group.

#### Staffing

Our staffing plan ensures coverage by study personnel for 8 weeks of the intervention and the 3- and 6-month follow-up assessment.

#### Lack of Skills to Use BBS Technology

This study does not expect low-income older adults to test themselves using the BBS. We will train an RA at each site to run the test.

### Conclusions

More than half of all older adults have maladaptive FRA, leading to reduced PA and a corresponding increased risk of falls. This study focuses on fall interventions tailored to low-income older adults who have a mismatch between physiological fall risk (body) and perceived fall risk (mind). The PEER is a novel intervention that combines concepts of physio-feedback, cognitive reframing, and peer-led exercise by motivating a shift in self-estimation of fall risk to align with physiological fall risk to improve balance, PA, and negative aging self-perception that no one has studied before. The major strength of this study is the use of a noninvasive technology that does not require special training and can operate in low-resource environments with unlimited users. The PEER intervention can be implemented on a large scale in community settings. Such an approach may reach older individuals at risk who do not participate in or are not referred to standardized conventional training programs to help them prevent falls and reduce health disparities.

## Abbreviations

BBS: BTrackS Balance Tracking System
CDC: Centers for Disease Control and Prevention
COREQ: Consolidated Criteria for Reporting Qualitative Research
FES-I: Falls Efficacy Scale-International
FOF: fear of falling
FRA: fall risk appraisalGLMM: Generalized Linear Mixed Model
ICC: intraclass correlation
LMM: linear mixed model
LRT: likelihood ratio test
MVPA: moderate to vigorous physical activity
PA: physical activity
PC: peer coach
PD: project director
PEER: Physio-Feedback Exercise Program
PI: principal investigator
RA: research assistant
REDCap: Research Electronic Data Capture
STS: Sit to Stand
TUG: Timed Up and Go

## Data Availability

The data sets generated during this study are available from the corresponding author upon reasonable request.

## References

[1] (2021). Poverty grew in 2020 as Americans lost income and health insurance. USA Facts.

[2] (2017). STEDI-older adult fall prevention. Centers for Disease Control and Prevention.

[3] Wehner-Hewson N, Watts P, Buscombe R, Bourne N, Hewson D (2022). Racial and ethnic differences in falls among older adults: a systematic review and meta-analysis. J Racial Ethn Health Disparities.

[4] Silva AD, Faleiros HH, Shimizu WA, Nogueira Lde M, Nhãn LL, Silva BA, Otuyama PM (2012). [The prevalence of falls and associated factors among the elderly according to ethnicity]. Cien Saude Colet.

[5] Chen TY, Kim G (2021). Racial/ethnic differences in the longitudinal effects of fear of falling on falls. Gerontology.

[6] Huang SH, Hsing SC, Sun CA, Chung CH, Tsao CH, Chung RJ, Wang BL, Huang YC, Chien WC (2021). Inequality in health: the correlation between poverty and injury-a comprehensive analysis based on income level in Taiwan: a cross-sectional study. Healthcare (Basel).

[7] Zandy M, Zhang LR, Kao D, Rajabali F, Turcotte K, Zheng A, Oakey M, Smolina K, Pike I, Rasali D (2019). Area-based socioeconomic disparities in mortality due to unintentional injury and youth suicide in British Columbia, 2009-2013. Health Promot Chronic Dis Prev Can.

[8] Berry SD, Miller RR (2008). Falls: epidemiology, pathophysiology, and relationship to fracture. Curr Osteoporos Rep.

[9] Florence CS, Bergen G, Atherly A, Burns E, Stevens J, Drake C (2018). Medical costs of fatal and nonfatal falls in older adults. J Am Geriatr Soc.

[10] Carey G, Crammond B (2015). Systems change for the social determinants of health. BMC Public Health.

[11] Stehr P, Luetke Lanfer H, Rossmann C (2021). Beliefs and motivation regarding physical activity among older adults in Germany: results of a qualitative study. Int J Qual Stud Health Well-being.

[12] Cho H, Seol SJ, Yoon DH, Kim MJ, Choi BY, Kim T (2013). Disparity in the fear of falling between urban and rural residents in relation with socio-economic variables, health issues, and functional independency. Ann Rehabil Med.

[13] Moser C, Spagnoli J, Santos-Eggimann B (2011). Self-perception of aging and vulnerability to adverse outcomes at the age of 65-70 years. J Gerontol B Psychol Sci Soc Sci.

[14] Sun JK, Smith J (2017). Self-perceptions of aging and perceived barriers to care: reasons for health care delay. Gerontologist.

[15] Choi K, Ko Y (2015). Characteristics associated with fear of falling and activity restriction in South Korean older adults. J Aging Health.

[16] Choi K, Jeon G, Cho S (2017). Prospective study on the impact of fear of falling on functional decline among community dwelling elderly women. Int J Environ Res Public Health.

[17] Plow MA, Allen SM, Resnik L (2011). Correlates of physical activity among low-income older adults. J Appl Gerontol.

[18] (2015). The United States of aging survey low-and moderate-income findings. United States of Aging Survey.

[19] Craike M, Bourke M, Hilland TA, Wiesner G, Pascoe MC, Bengoechea EG, Parker AG (2019). Correlates of physical activity among disadvantaged groups: a systematic review. Am J Prev Med.

[20] Gaskin CJ, Orellana L (2018). Factors associated with physical activity and sedentary behavior in older adults from six low- and middle-income countries. Int J Environ Res Public Health.

[21] Su TT, Azzani M, Adewale AP, Thangiah N, Zainol R, Majid H (2019). Physical activity and health-related quality of life among low-income adults in metropolitan Kuala Lumpur. J Epidemiol.

[22] Gunn H, Cameron M, Hoang P, Lord S, Shaw S, Freeman J (2018). Relationship between physiological and perceived fall risk in people with multiple sclerosis: implications for assessment and management. Arch Phys Med Rehabil.

[23] Thiamwong L (2020). A hybrid concept analysis of fall risk appraisal: Integration of older adults' perspectives with an integrative literature review. Nurs Forum.

[24] Thiamwong L, Ng BP, Kwan RY, Suwanno J (2021). Maladaptive fall risk appraisal and falling in community-dwelling adults aged 60 and older: implications for screening. Clin Gerontol.

[25] Ng BP, Thiamwong L, He Q, Towne SD Jr, Li Y (2020). Discrepancies between perceived and physiological fall risks and repeated falls among community-dwelling medicare beneficiaries aged 65 years and older. Clin Gerontol (Forthcoming).

[26] Thiamwong L, Sole ML, Ng BP, Welch GF, Huang HJ, Stout JR (2020). Assessing fall risk appraisal through combined physiological and perceived fall risk measures using innovative technology. J Gerontol Nurs.

[27] Delbaere K, Close JC, Brodaty H, Sachdev P, Lord SR (2010). Determinants of disparities between perceived and physiological risk of falling among elderly people: cohort study. BMJ.

[28] Yoshikawa A, Ramirez G, Smith ML, Lee S, Ory MG (2020). Systematic review and meta-analysis of fear of falling and fall-related efficacy in a widely disseminated community-based fall prevention program. Arch Gerontol Geriatr.

[29] Sakurai R, Fujiwara Y, Yasunaga M, Suzuki H, Sakuma N, Imanaka K, Montero-Odasso M (2017). Older adults with fear of falling show deficits in motor imagery of gait. J Nutr Health Aging.

[30] Delbaere K, Crombez G, Van Den Noortgate N, Willems T, Cambier D (2006). The risk of being fearful or fearless of falls in older people: an empirical validation. Disabil Rehabil.

[31] Thiamwong L, Xie R, Choudhury R, Park JH, Garcia O, Rossler A, Stout J (2021). Associations among fall risk appraisal, body composition, and physical activity in older adults. Innovation in Aging.

[32] Thiamwong L, Stout JR, Sole ML, Ng BP, Yan X, Talbert S (2020). Physio-feedback and exercise program (PEER) improves balance, muscle strength, and fall risk in older adults. Res Gerontol Nurs.

[33] Kendrick D, Kumar A, Carpenter H, Zijlstra GA, Skelton DA, Cook JR, Stevens Z, Belcher CM, Haworth D, Gawler SJ, Gage H, Masud T, Bowling A, Pearl M, Morris RW, Iliffe S, Delbaere K (2014). Exercise for reducing fear of falling in older people living in the community. Cochrane Database Syst Rev.

[34] Whipple MO, Hamel AV, Talley KM (2018). Fear of falling among community-dwelling older adults: a scoping review to identify effective evidence-based interventions. Geriatr Nurs.

[35] Cox TB, Williams K (2016). Fall recovery intervention and its effect on fear of falling in older adults. Act Adapt Aging.

[36] Iaboni A, Banez C, Lam R, Jones SA, Maki BE, Liu BA, Flint AJ (2015). Depression and outcome of fear of falling in a falls prevention program. Am J Geriatr Psychiatry.

[37] Kumar A, Delbaere K, Zijlstra GA, Carpenter H, Iliffe S, Masud T, Skelton D, Morris R, Kendrick D (2016). Exercise for reducing fear of falling in older people living in the community: cochrane systematic review and meta-analysis. Age Ageing.

[38] Robinson JB, Wetherell JL (2018). An interdisciplinary intervention for fear of falling: lessons learned from two case studies. Clin Gerontol.

[39] Tennstedt S, Howland J, Lachman M, Peterson E, Kasten L, Jette A (1998). A randomized, controlled trial of a group intervention to reduce fear of falling and associated activity restriction in older adults. J Gerontol B Psychol Sci Soc Sci.

[40] Wetherell JL, Johnson K, Chang D, Ward SR, Bower ES, Merz C, Petkus AJ (2016). Activity, balance, learning, and exposure (ABLE): a new intervention for fear of falling. Int J Geriatr Psychiatry.

[41] Zijlstra GA, van Haastregt JC, Ambergen T, van Rossum E, van Eijk JT, Tennstedt SL, Kempen GI (2009). Effects of a multicomponent cognitive behavioral group intervention on fear of falling and activity avoidance in community-dwelling older adults: results of a randomized controlled trial. J Am Geriatr Soc.

[42] Litwin H, Erlich B, Dunsky A (2018). The complex association between fear of falling and mobility limitation in relation to late-life falls: a SHARE-based analysis. J Aging Health.

[43] Kumar A, Carpenter H, Morris R, Iliffe S, Kendrick D (2014). Which factors are associated with fear of falling in community-dwelling older people?. Age Ageing.

[44] Hamm J, Money AG, Atwal A, Paraskevopoulos I (2016). Fall prevention intervention technologies: a conceptual framework and survey of the state of the art. J Biomed Inform.

[45] Oh-Park M, Doan T, Dohle C, Vermiglio-Kohn V, Abdou A (2021). Technology utilization in fall prevention. Am J Phys Med Rehabil.

[46] Valenzuela T, Okubo Y, Woodbury A, Lord SR, Delbaere K (2018). Adherence to technology-based exercise programs in older adults: a systematic review. J Geriatr Phys Ther.

[47] Levy SS, Thralls KJ, Kviatkovsky SA (2018). Validity and reliability of a portable balance tracking system, btracks, in older adults. J Geriatr Phys Ther.

[48] O'Connor SM, Baweja HS, Goble DJ (2016). Validating the BTrackS Balance Plate as a low cost alternative for the measurement of sway-induced center of pressure. J Biomech.

[49] Lachman ME, Weaver SL, Bandura M, Elliott E, Lewkowicz CJ (1992). Improving memory and control beliefs through cognitive restructuring and self-generated strategies. J Gerontol.

[50] Matz-Costa C, Howard EP, Castaneda-Sceppa C, Diaz-Valdes Iriarte A, Lachman ME (2019). Peer-based strategies to support physical activity interventions for older adults: a typology, conceptual framework, and practice guidelines. Gerontologist.

[51] Thiamwong L, Park, JH, Choudhury R, Garcia O, Furtado M, Stallworth N, Stout J (2021). Using assistive health technology to assess fall risk appraisal, body composition, and physical activity. Innov Aging.

[52] Thiamwong L, Garcia O, Choudhury R, Park JH, Stout J, Xie R (2021). Feasibility and acceptability of the technology-based fall risk assessments for older adults. Innov Aging.

[53] Melix BL, Uejio CK, Kintziger KW, Reid K, Duclos C, Jordan MM, Holmes T, Joiner J (2020). Florida neighborhood analysis of social determinants and their relationship to life expectancy. BMC Public Health.

[54] (2018). Qickfacts Florida: persons 65 years and overs (percent). U.S. and Census.

[55] Folstein MF, Folstein SE, McHugh PR (1975). "Mini-mental state". A practical method for grading the cognitive state of patients for the clinician. J Psychiatr Res.

[56] Nyman SR, Victor CR (2012). Older people's participation in and engagement with falls prevention interventions in community settings: an augment to the Cochrane systematic review. Age Ageing.

[57] Newton RL Jr, Carter LA, Johnson W, Zhang D, Larrivee S, Kennedy BM, Harris M, Hsia DS (2018). A church-based weight loss intervention in African American adults using text messages (LEAN Study): cluster randomized controlled trial. J Med Internet Res.

[58] Mazzeo RS (1998). American college of sports medicine position stand. Exercise and physical activity for older adults. Med Sci Sports Exerc.

[59] Chodzko-Zajko WJ, Proctor DN, Fiatarone Singh MA, Minson CT, Nigg CR, Salem GJ, Skinner JS, American College of Sports Medicine (2009). American college of sports medicine position stand. Exercise and physical activity for older adults. Med Sci Sports Exerc.

[60] Lesinski M, Hortobágyi T, Muehlbauer T, Gollhofer A, Granacher U (2015). Effects of balance training on balance performance in healthy older adults: a systematic review and meta-analysis. Sports Med.

[61] Borrelli B, Sepinwall D, Ernst D, Bellg AJ, Czajkowski S, Breger R, DeFrancesco C, Levesque C, Sharp DL, Ogedegbe G, Resnick B, Orwig D (2005). A new tool to assess treatment fidelity and evaluation of treatment fidelity across 10 years of health behavior research. J Consult Clin Psychol.

[62] Borrelli B (2011). The assessment, monitoring, and enhancement of treatment fidelity in public health clinical trials. J Public Health Dent.

[63] Moore GF, Audrey S, Barker M, Bond L, Bonell C, Hardeman W, Moore L, O'Cathain A, Tinati T, Wight D, Baird J (2015). Process evaluation of complex interventions: medical Research Council guidance. BMJ.

[64] Nithman RW, Vincenzo JL (2019). How steady is the STEADI? Inferential analysis of the CDC fall risk toolkit. Arch Gerontol Geriatr.

[65] Zasadzka E, Borowicz AM, Roszak M, Pawlaczyk M (2015). Assessment of the risk of falling with the use of timed up and go test in the elderly with lower extremity osteoarthritis. Clin Interv Aging.

[66] Ory MG, Smith ML, Jiang L, Lee R, Chen S, Wilson AD, Stevens JA, Parker EM (2014). Fall prevention in community settings: results from implementing stepping on in three States. Front Public Health.

[67] Shumway-Cook A, Silver IF, LeMier M, York S, Cummings P, Koepsell TD (2007). Effectiveness of a community-based multifactorial intervention on falls and fall risk factors in community-living older adults: a randomized, controlled trial. J Gerontol A Biol Sci Med Sci.

[68] Bohannon RW (2006). Reference values for the timed up and go test: a descriptive meta-analysis. J Geriatr Phys Ther.

[69] Shumway-Cook A, Brauer S, Woollacott M (2000). Predicting the probability for falls in community-dwelling older adults using the Timed Up and Go Test. Phys Ther.

[70] Jones CJ, Rikli RE, Beam WC (1999). A 30-s chair-stand test as a measure of lower body strength in community-residing older adults. Res Q Exerc Sport.

[71] Bohannon RW, Crouch R (2019). 1-Minute Sit-to-Stand Test: systematic review of procedures, performance, and clinimetric properties. J Cardiopulm Rehabil Prev.

[72] Podsiadlo D, Richardson S (1991). The Timed Up and Go: a test of basic functional mobility for frail elderly persons. J Am Geriatr Soc.

[73] Bischoff HA, Stähelin HB, Monsch AU, Iversen MD, Weyh A, von Dechend M, Akos R, Conzelmann M, Dick W, Theiler R (2003). Identifying a cut-off point for normal mobility: a comparison of the timed 'up and go' test in community-dwelling and institutionalised elderly women. Age Ageing.

[74] Gao KL, Tsang WW (2008). Use of accelerometry to quantify the physical activity level of the elderly. Hong Kong Physiother J.

[75] Hornyak V, Brach JS, Wert DM, Hile E, Studenski S, VanSwearingen JM (2013). What is the relation between fear of falling and physical activity in older adults?. Arch Phys Med Rehabil.

[76] Heesch KC, Hill RL, Aguilar-Farias N, van Uffelen JG, Pavey T (2018). Validity of objective methods for measuring sedentary behaviour in older adults: a systematic review. Int J Behav Nutr Phys Act.

[77] Thiamwong L, Huang HJ, Ng BP, Yan X, Sole ML, Stout JR, Talbert S (2020). Shifting maladaptive fall risk appraisal in older adults through an in-home Physio-fEedback and Exercise pRogram (PEER): a pilot study. Clin Gerontol.

[78] Delbaere K, Close JC, Mikolaizak AS, Sachdev PS, Brodaty H, Lord SR (2010). The Falls Efficacy Scale International (FES-I). A comprehensive longitudinal validation study. Age Ageing.

[79] Yardley L, Beyer N, Hauer K, Kempen G, Piot-Ziegler C, Todd C (2005). Development and initial validation of the Falls Efficacy Scale-International (FES-I). Age Ageing.

[80] Kempen GI, Yardley L, van Haastregt JC, Zijlstra GAR, Beyer N, Hauer K, Todd C (2008). The Short FES-I: a shortened version of the falls efficacy scale-international to assess fear of falling. Age Ageing.

[81] del-Río-Valeiras M, Gayoso-Diz P, Santos-Pérez S, Rossi-Izquierdo M, Faraldo-García A, Vaamonde-Sánchez-Andrade I, Lirola-Delgado A, Soto-Varela A (2016). Is there a relationship between short FES-I test scores and objective assessment of balance in the older people with age-induced instability?. Arch Gerontol Geriatr.

[82] Marques-Vieira CM, Sousa LM, Severino SS, Sousa L, Caldeira S (2016). Cross-cultural validation of the falls efficacy scale international in elderly: systematic literature review. J Clin Gerontol Geriatr.

[83] Valentine JD, Simpson J, Worsfold C, Fisher K (2011). A structural equation modelling approach to the complex path from postural stability to morale in elderly people with fear of falling. Disabil Rehabil.

[84] Goble DJ, Baweja HS (2018). Postural sway normative data across the adult lifespan: results from 6280 individuals on the Balance Tracking System balance test. Geriatr Gerontol Int.

[85] Fawsitt F, Dockray S, Setti A (2022). Regulatory focus and perceptions of ageing: exploring the connections. Aging Ment Health.

[86] Freeman AT, Santini ZI, Tyrovolas S, Rummel-Kluge C, Haro JM, Koyanagi A (2016). Negative perceptions of ageing predict the onset and persistence of depression and anxiety: findings from a prospective analysis of the Irish Longitudinal Study on Ageing (TILDA). J Affect Disord.

[87] Social determinants of health: core. PhenX Toolkit.

[88] Phelan E, Williams B, Meeker K, Bonn K, Frederick J, Logerfo J, Snowden M (2010). A study of the diagnostic accuracy of the PHQ-9 in primary care elderly. BMC Fam Pract.

[89] Familiar I, Ortiz-Panozo E, Hall B, Vieitez I, Romieu I, Lopez-Ridaura R, Lajous M (2015). Factor structure of the Spanish version of the Patient Health Questionnaire-9 in Mexican women. Int J Methods Psychiatr Res.

[90] Balsamo M, Cataldi F, Carlucci L, Fairfield B (2018). Assessment of anxiety in older adults: a review of self-report measures. Clin Interv Aging.

[91] Byrne GJ, Pachana NA (2011). Development and validation of a short form of the Geriatric Anxiety Inventory--the GAI-SF. Int Psychogeriatr.

[92] Márquez-González M, Losada A, Fernández-Fernández V, Pachana NA (2012). Psychometric properties of the Spanish version of the Geriatric Anxiety Inventory. Int Psychogeriatr.

[93] Johnco C, Knight A, Tadic D, Wuthrich VM (2015). Psychometric properties of the Geriatric Anxiety Inventory (GAI) and its short-form (GAI-SF) in a clinical and non-clinical sample of older adults. Int Psychogeriatr.

[94] Ward RE, Leveille SG, Beauchamp MK, Travison T, Alexander N, Jette AM, Bean JF (2015). Functional performance as a predictor of injurious falls in older adults. J Am Geriatr Soc.

[95] Welmer AK, Rizzuto D, Laukka EJ, Johnell K, Fratiglioni L (2017). Cognitive and physical function in relation to the risk of injurious falls in older adults: a population-based study. J Gerontol A Biol Sci Med Sci.

[96] Maidan I, Freedman T, Tzemah R, Giladi N, Mirelman A, Hausdorff JM (2014). Introducing a new definition of a near fall: intra-rater and inter-rater reliability. Gait Posture.

[97] (2018). Research electronic data capture. REDCap.

[98] Harris PA, Taylor R, Thielke R, Payne J, Gonzalez N, Conde JG (2009). Research electronic data capture (REDCap)--a metadata-driven methodology and workflow process for providing translational research informatics support. J Biomed Inform.

[99] Jiang K, Cao X (2011). Design and implementation of an audit trail in compliance with US regulations. Clin Trials.

[100] McCoy E (2017). Understanding the intention-to-treat principle in randomized controlled trials. West J Emerg Med.

[101] Liu S, Rovine MJ, Molenaar PC (2012). Selecting a linear mixed model for longitudinal data: repeated measures analysis of variance, covariance pattern model, and growth curve approaches. Psychol Methods.

[102] (2021). Assessment 30-second chair stand. Centers for Disease Control and Prevention.

[103] Migueles JH, Rowlands AV, Huber F, Sabia S, van Hees VT (2019). GGIR: a research community–driven open source r package for generating physical activity and sleep outcomes from multi-day raw accelerometer data. J Meas Phys Behav (Forthcoming).

[104] van Hees VT, Fang Z, Langford J, Assah F, Mohammad A, da Silva IC, Trenell MI, White T, Wareham NJ, Brage S (2014). Autocalibration of accelerometer data for free-living physical activity assessment using local gravity and temperature: an evaluation on four continents. J Appl Physiol (1985).

[105] van Hees VT, Gorzelniak L, Dean León EC, Eder M, Pias M, Taherian S, Ekelund U, Renström F, Franks PW, Horsch A, Brage S (2013). Separating movement and gravity components in an acceleration signal and implications for the assessment of human daily physical activity. PLoS One.

[106] Enders CK, Mistler SA, Keller BT (2016). Multilevel multiple imputation: a review and evaluation of joint modeling and chained equations imputation. Psychol Methods.

[107] Cohen J (1988). Statistical Power Analysis for the Behavioral Sciences. 2nd edition.

[108] Lakens D (2013). Calculating and reporting effect sizes to facilitate cumulative science: a practical primer for t-tests and ANOVAs. Front Psychol.

[109] Hardin JW, Hilbe JM (2003). Generalized Estimating Equations.

[110] Padgett DK (2011). Qualitative and Mixed Methods in Public Health.

[111] Thorne S (2016). Interpretive Description: Qualitative Research for Applied Practice. 2nd edition.

[112] Guest G, Bunce A, Johnson L (2006). How many interviews are enough?: An experiment with data saturation and variability. Field Methods.

[113] Tong A, Sainsbury P, Craig J (2007). Consolidated criteria for reporting qualitative research (COREQ): a 32-item checklist for interviews and focus groups. Int J Qual Health Care.

[114] Padgett DK (2017). Qualitative Methods in Social Work Research. 3rd edition.

[115] (2017). NIMHD minority health and health disparities research framework. National Institute on Minority Health and Health Disparities.

[116] Thiamwong L (2021). Older adults' experiences with the visual physio-feedback technology and peer-led combined group and home-based exercises. J Aging Phys Act.

[117] Thorne S, Kirkham SR, MacDonald-Emes J (1997). Interpretive description: a noncategorical qualitative alternative for developing nursing knowledge. Res Nurs Health.

[118] Thorne S, Kirkham SR, O'Flynn-Magee K (2016). The analytic challenge in interpretive description. Int J Qual Methods.

[119] Hesse-Biber S (2012). Weaving a multimethodology and mixed methods praxis into randomized control trials to enhance credibility. Qual Inq.

[120] Clark VL, Ivankova NV (2016). Mixed Methods Research: A Guide to the Field.

[121] Craike M, Wiesner G, Hilland TA, Bengoechea EG (2018). Interventions to improve physical activity among socioeconomically disadvantaged groups: an umbrella review. Int J Behav Nutr Phys Act.

[122] (2018). Physical activity guidelines for Americans. US Department of Health and Human Services.

